# High serum levels of leucine-rich α-2 glycoprotein 1 (LRG-1) are associated with poor survival in patients with early breast cancer

**DOI:** 10.1007/s00404-024-07434-0

**Published:** 2024-02-28

**Authors:** Andy Göbel, Tilman D. Rachner, Oliver Hoffmann, Daniel Martin Klotz, Sabine Kasimir-Bauer, Rainer Kimmig, Lorenz C. Hofbauer, Ann-Kathrin Bittner

**Affiliations:** 1 Division of Endocrinology and Metabolic Bone Diseases, Department of Medicine III, Technische Universität Dresden, Dresden, Germany; 2 Center for Healthy Ageing Department of Medicine III, Technische Universität Dresden, Dresden, Germany; 3 German Cancer Consortium (DKTK), Dresden, Germany; 4 German Cancer Research Center (DKFZ), Heidelberg, Germany; 5 Department of Gynecology and Obstetrics, University Hospital Essen, University of Duisburg-Essen, Essen, Germany; 6 National Center for Tumor Diseases (NCT), NCT West, Heidelberg, Germany; 7 Department of Gynecology and Obstetrics, Medical Faculty and University Hospital Carl Gustav Carus, Technische Universität Dresden, Dresden, Germany; 8 National Center for Tumor Diseases (NCT), Dresden, Germany; 9 Helmholtz-Zentrum Dresden - Rossendorf (HZDR), Dresden, Germany

**Keywords:** LRG-1; Early breast cancer; Prognostic marker; Serum marker; Minimal residual disease; Disseminated tumor cells

## Abstract

**Background:**

Leucine-rich α-2 glycoprotein 1 (LRG-1) is a secreted glycoprotein that is mainly produced in the liver. Elevated levels of LRG-1 are found in a multitude of pathological conditions including eye diseases, diabetes, infections, autoimmune diseases, and cancer. In patients with early breast cancer (BC), high intratumoral LRG-1 protein expression levels are associated with reduced survival. In this study, we assessed serum levels of LRG-1 in patients with early BC and investigated its correlation with the presence of disseminated tumor cells (DTCs) in the bone marrow and survival outcomes.

**Methods:**

Serum LRG-1 levels of 509 BC patients were determined using ELISA and DTCs were assessed by immunocytochemistry using the pan-cytokeratin antibody A45-B/B3. We stratified LRG-1 levels according to selected clinical parameters. Using the log-rank (Mantel–Cox) test and multivariate Cox regression analysis, Kaplan–Meier survival curves and prognostic relevance were assessed.

**Results:**

Mean serum levels of LRG-1 were 29.70 ± 8.67 µg/ml. Age was positively correlated with LRG-1 expression (r = 0.19; p < 0.0001) and significantly higher LRG-1 levels were found in patients over 60 years compared to younger ones (30.49 ± 8.63 µg/ml vs. 28.85 ± 8.63 µg/ml; p = 0.011) and in postmenopausal patients compared to premenopausal patients (30.15 ± 8.34 µg/ml vs. 26.936.94 µg/ml; p = 0.002). Patients with no DTCs showed significantly elevated LRG-1 levels compared to the DTC-positive group (30.51 ± 8.69 µg/ml vs. 28.51 ± 8.54 µg/ml; p = 0.004). Overall and BC-specific survival was significantly lower in patients with high serum LRG-1 levels (above a cut-off of 33.63 µg/ml) compared to patients with lower LRG-1 levels during a mean follow-up of 8.5 years (24.8% vs. 11.1% BC-specific death; p = 0.0003; odds ratio 2.63, 95%CI: 1.56—4.36). Multivariate analyses revealed that LRG-1 is an independent prognostic marker for BC-specific survival (p = 0.001; hazard ratio 2.61).

**Conclusions:**

This study highlights the potential of LRG-1 as an independent prognostic biomarker in patients with early BC.

## What does this study add to the clinical work

**Table:** 

Our study reveals that serum leucine-rich α-2 glycoprotein 1 (LRG-1) is an independent prognostic marker for breast cancer-specific survival. This finding might translate into novel diagnostic approaches especially as the analysis of serum samples in patients with cancer are more readily available, cost-effective and can be easily implemented into routine clinical diagnostics.	

## Background

Leucine-rich α-2 glycoprotein 1 (LRG-1) is a secreted glycoprotein that contains the evolutionary conserved leucine-rich repeat (LRR) motif [1]. Members of the LRR protein family exist in a broad range of organisms including plants, bacteria, animals, and fungi where they hold essential functions in innate immunity and protein–protein interactions, among others [2, 3]. LRG-1 is mainly expressed by hepatocytes and differentiating neutrophils and can be stimulated by a spectrum of inflammatory factors such as lipopolysaccharide, interleukin (IL)-1β, IL-6, and tumor necrosis factor alpha (TNF-α), initially pointing towards a potential role as an important mediator of acute phase reactions [4–6]. The physiological role of LRG-1 is not fully understood yet, especially as LRG-1 knockout mice are viable and present only with a mild phenotype [7]. LRG-1 is described to be locally and systemically upregulated in disease [2]. In murine models of retinal vascular pathology, LRG-1 is overexpressed in the vasculature of the retina and contributes to pathogenic angiogenesis by modulating transforming growth factor beta signaling [7]. In patients with age-related macular degeneration and diabetic retinopathy, LRG-1 levels are increased in the serum, the endothelia of abnormally formed blood vessels in the retina, and in the aqueous humor [8, 9]. By interfering with either the communication between endothelial cells and pericytes or by direct activation of endothelial cells, LRG-1 facilitates the formation of highly permeable, disorganized new blood vessels [10]. Similar mechanisms of pathological neovascularization by upregulated LRG-1 are seen in diabetic and chronic kidney disease [11, 12].

Studies from inflammatory diseases have elucidated that LRG-1 supports pro-inflammatory immune reactions as well as the differentiation and recruitment of neutrophils [2]. In rheumatoid arthritis, Crohn’s disease and ulcerative colitis, serum LRG-1 levels are increased and correlate with disease activity [13, 14]. Other pathologies in which LRG-1 is reported to be involved include infectious, metabolic, cardiovascular, nervous system-related diseases and chronic wound formation and fibrosis [2, 12, 15–17].

LRG-1 plays a role in inflammation, fibrosis, metabolism, and angiogenesis, all of which contribute to tumor initiation and progression. Consequently, an increasing number of studies have reported an aberrant activation and functional role of LRG-1 in cancer. Here, LRG-1 from different cellular sources promotes pathological vessel formation, immunosuppression, epithelial-to-mesenchymal transition, metastasis and tumor cell proliferation [18–23]. In addition, tumor tissue and serum levels of LRG-1 are increased and associated with worse prognosis in numerous malignancies such as melanoma, colorectal, pancreatic, prostate, lung, hepatocellular, and ovarian cancer [18, 20, 21, 24–33].

In breast cancer (BC), LRG-1 has anti-apoptotic potential and its increased tumor tissue expression is associated with worse prognosis and lymph node metastasis [34, 35]. However, the prognostic potential of serum LRG-1 in patients with early BC remains unknown. In this study, we aimed at addressing this question by using a well-defined patient cohort, comparing LRG-1 with clinicopathological markers as well as with the presence of disseminated tumor cells (DTCs). DTCs are a micrometastatic tumor spread to the bone marrow (BM), surviving in a state of dormancy [36], called minimal residual disease [37, 38]. DTCs are an independent prognostic marker for BC overall survival (OS), disease-free survival and distant disease-free survival [39]. We therefore also assessed potential correlations between serum LRG-1 levels and the presence of DTCs in patients with early BC.

## Patients and methods

### Patient population and study design

We used a cohort that has been previously described in published biomarker studies [40, 41] and report on this cohort again according to the REMARK guidelines [42]. The cohort consists of 509 patients with primary, early BC diagnosed between 2004 and 2009. Using protocols approved by the clinical ethics committee of the University Hospital Essen (05/2856), serum samples were collected before systemic therapy/at the beginning of surgery after written informed consent was obtained from all patients. The eligibility criteria for the inclusion were: histologically proven BC, no severe uncontrolled co-morbidities or medical conditions, BM aspiration at the time of primary diagnosis, no neoadjuvant chemotherapy and no present or previous other malignancies and metastasis. Therapeutic treatment followed current national guidelines [43] including adjuvant chemotherapy (anthracyclines, 5-fluorouracil, taxanes, and cyclophosphamide), anti-hormonal therapy in the case of hormone-responsive tumors (tamoxifen or an aromatase inhibitor), trastuzumab in the case of HER2-positivity (after FDA approval in November 2006) and radiotherapy, if indicated. Grading, TNM-staging and assessment of tumor type were performed at the Institute of Pathology of the University Hospital Essen as part of the West German Comprehensive Cancer Center.

### Selection and detection of DTCs

DTC status was determined in all patients included in this study as previously described [41]. Briefly, BM aspirates (10–20 ml) were obtained from the anterior iliac crests of all patients at the beginning of surgery of the primary tumor and processed within 24 h. DTC isolation and detection was performed based on the recommendations for standardized tumor cell detection that have been published by the German Consensus group of Senology [44]. For the staining, cells were isolated from heparinized bone marrow (5000 U/ml BM) by Ficoll-Hypaque density gradient centrifugation (density 1.077 g/mol; Pharmacia, Freiburg, Germany) at 400 × g for 30 min. Using immunocytochemical staining with the pan-cytokeratin antibody A45-B/B3, slides were assessed for the presence of DTCs. Microscopic evaluation of the slides was performed according to the ISHAGE evaluation criteria using the ARIOL system (Applied Imaging).

### Sampling of serum

Prior to surgery of each patient, nine milliliters of blood were collected using an S-Monovette (Sarstedt AG & Co). Samples were immediately stored at 4 °C. To avoid blood cell lysis, samples were processed within 4 h. The fractionation of the blood was performed by centrifugation for ten minutes at 2,500 × g. Afterwards, 3 to 4 ml of blood serum (upper phase of the fractions) were removed. Serum samples were frozen at −80 °C until performing the ELISAs.

### Detection of LRG-1 by ELISA

LRG-1 serum levels were detected by ELISA (Biomedica, Vienna, Austria) according to the manufacturer’s instructions. Briefly, samples were diluted one to 4,000 with assay buffer and subsequently added to the wells. Following 2 h of incubation at room temperature, wells were washed five times using washing buffer. Now, 100 µl of antibody conjugate were added to each well and the preparation incubated for 1 h. Substrate was added after another washing step for five times and the plate incubated for 30 min at room temperature in the dark. Fifty µl of stop solution were added and the absorbance was measured immediately at 450 nm with reference at 630 nm using FLUOstar Omega (BMG Labtech, Ortenberg, Germany).

### Statistical analysis

The statistical analysis was conducted with R, Version 4.0.2 and GraphPad Prism version 9.0.0 (GraphPad Software, La Jolla, CA, USA) as described previously [40, 41], and listed in each figure legend. Results are presented as the mean ± 95% confidence interval (CI), unless otherwise stated. Comparison of two groups was assessed using the non-parametric, two‐sided Mann–Whitney test. Groups of three were assessed by ANOVA. The correlation was assessed by non-parametric Spearman correlation. Cut-off analysis was performed using maximally selected rank statistics (maxstat package). Kaplan–Meier analyses were performed with significance levels indicated by log-rank (Mantel–Cox) analysis and hazard ratios (HRs; Mantel–Haenszel) are shown with 95%CI. BC-specific survival was defined as time between diagnosis of the primary tumor and death directly related to the disease. Uni‐ and multivariate Cox proportional hazards model regression analyses were performed and HRs are indicated with 95%CI. P values < 0.05 were considered statistically significant.

## Results

### Cohort

Table 1 lists the main clinical characteristics of the patients at the time of initial diagnosis. The median age of patients included was 60.8 years and ranged from 27 to 86 years. Seventy-two patients (14.1%) were premenopausal, 374 were postmenopausal (73.5%) and 63 were considered perimenopausal (12.4%). Ductal breast carcinomas were found to be the predominant histological subtype (385/509; 75.6%). Of the different tumor stages ranging from pT1 to pT4, most women presented with pT1 tumors (321/509; 63.1%). Half of the cohort had a moderately differentiated tumor (270/509, 53%) and the majority of the cohort (340/509, 66.8%) were lymph-node negative. Stratification according to immunohistochemical subtype revealed that the majority of the patients was ER- and/or PR-positive and HER2-negative patients (364/509; 71.5%), 11.2% were triple-positive (57/509), 65 patients were triple-negative (12.8%) and 4.5% only showed HER2 overexpression (23/509). Two hundred and seven patients presented with a positive DTC status (207/509; 40.7%). Survival data were available for 504/509 patients (5 patients lost to follow-up) and the median follow-up was 8.5 years (range 0.16–13.64). Of the 76 patients who died, 74 (97.4%) specifically died from breast cancer.

**Table 1:** **Clinical baseline characteristics of patients**. Patient characteristics are presented as total number (n) and percentage of all (%)

	**Total (%)**
**Total**	509
**Age (years)**	
Median	60.8 (range 27 to 86 years)
**Menopausal status**	
Premenopausal Perimenopausal Postmenopausal	72 (14.1) 63 (12.4) 374 (73.5)
**Histology**	
Ductal Lobular Others	385 (75.6) 68 (13.4) 56 (11.0)
**Tumor stage**	
pT1 pT2 pT3–4 pTis Unknown	321 (63.1) 160 (31.4) 24 (4.7) 3 (0.6) 1 (0.2)
**Nodal status**	
Node negative Node positive Unknown	340 (66.8) 167 (32.8) 2 (0.4)
**Grading**	
I II III Unknown	89 (17.5) 270 (53.0) 149 (29.3) 1 (0.2)
**ER status**	
Negative Positive Unknown	94 (18.5) 414 (81.3) 1 (0.2)
**PR status**	
Negative Positive Unknown	135 (26.5) 373 (73.3) 1 (0.2)
**Her2 status**	
Negative Positive Unknown	426 (83.7) 80 (15.7) 3 (0.6)
**Immunohistochemical subtype**	
(ER-, PR-, Her2-) (ER-, PR-, Her2 +) (ER + and/ or PR + , Her2-) (ER + and/ or PR + , Her2 +)	65 (12.8) 23 (4.5) 364 (71.5) 57 (11.2)
**DTC status**	
Positive Negative Unknown	207 (40.7) 300 (58.9) 2 (0.4)

Adapted and modified from Rachner et al. (2018) and Rachner et al. (2020)

### High LRG-1 correlated with clinicopathological parameters

**Fig. 1:**
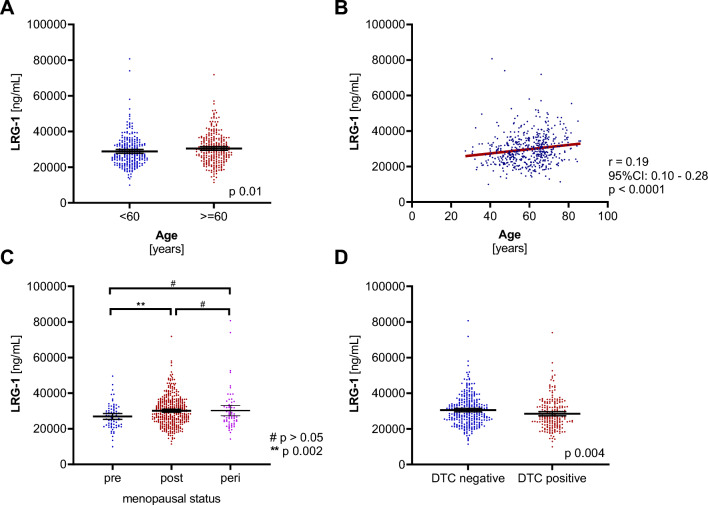
**High LRG-1 correlated with clinicopathological parameters**. **a** Scatter plots comparing LRG-1 levels in breast cancer patients by age with 60 years as cut-off. **b** The correlation of LRG-1 and age, using non-parametric Spearman correlation (*n* = 506) with simple linear regression (red line). **c** Scatter plots comparing LRG-1 levels in pre-, post and perimenopausal breast cancer patients. **d** Scatter plots comparing LRG-1 levels in patients with DTC and without DTC (DTC positive and DTC negative). The black horizontal lines indicate median LRG-1 levels in each group, with error bars showing the 95%CI. P-value according to the non-parametric, two‐sided Mann–Whitney test

**Fig. 2:**
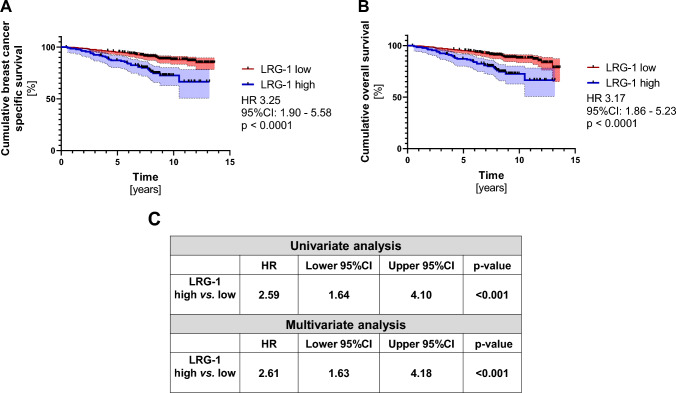
**High LRG-1 levels are associated with a poor prognosis and is an independent prognostic marker.** Kaplan–Meier analysis comparing **a** cumulative breast cancer-specific survival and **b** cumulative overall survival of LRG-1 high (*n* = 133) versus LRG-1 low (*n* = 368) breast cancer patients. HR and 95% CI according to Mantel Haenszel and p-value according to log-rank (Mantel–Cox) are indicated. **c** The univariate and multivariate analysis is shown for patients with high LRG-1 versus patients with low LRG-1 serum levels. HR, 95%CI and p-values are shown. The cut-off (33.64 µg/ml) is determined as described in the methods section

We quantified serum levels of LRG-1 in all patients. Valid LRG-1 levels were recorded in 99.4% (506/509) of the samples. Mean serum levels of LRG-1 were 29.70 ± 8.67 µg/ml in this patient cohort. Table 2 shows LRG-1 levels at baseline when stratifying the cohort according to age, menopausal status, histology, tumor stage, nodal status, grading, hormone receptor status, and DTCs status. In patients older than 60 years, LRG-1 levels were significantly increased compared to younger patients (30.49 ± 8.63 µg/ml vs. 28.85 ± 8.63 µg/ml; p = 0.011; Fig. 1a). In addition, age was positively correlated with serum LRG-1 levels (*r* = 0.19; 95% CI: 0.10 – 0.28; *p* < 0.0001; Fig. 2b). Moreover, LRG-1 serum levels in postmenopausal patients were significantly increased compared to premenopausal patients (30.15 ± 8.34 µg/ml vs. 26.93 ± 6.94 µg/ml; *p* = 0.002; Fig. 1c). Of note, an increased LRG-1 concentration was found in patients with no detectable DTCs compared to those present with DTCs positivity (30.51 ± 8.69 µg/ml vs. 28.51 ± 8.54 µg/ml; *p* = 0.004; Fig. 1d). Neither tumor histology and stage, grading, nodal status, nor hormone receptor expression status did affect circulating LRG-1 levels.

**Table 2:** **LRG-1 serum levels in breast cancer patients**. Values of LRG-1 are given in µg/ml and represent mean ± standard deviation. Comparison of two groups was assessed using the non-parametric, two‐sided Mann–Whitney test. Groups of three were assessed by ANOVA

	**LRG-1 (µg/ml)**	**p value**
**Age (years)**		
< 60 > 60	28.85 ± 8.63 30.49 ± 8.63	0.011
**Menopausal status**		
Premenopausal	26.93 ± 6.94	
Perimenopausal	30.21 ± 11.36	
Postmenopausal	30.15 ± 8.34	(0.002 vs pre.)
**Histology**		
Ductal	29.84 ± 8.85	ns
Lobular	27.99 ± 7.74	
Others	30.80 ± 8.23	
**Tumor stage**		
pT1	29.37 ± 8.09	ns
pT2	29.85 ± 9.62	
pT3–4	32.68 ± 8.95	
**Nodal status**		
Node negative	29.36 ± 8.70	ns
Node positive	30.02 ± 8.47	
**Grading**		
I	30.89 ± 7.98	ns
II	29.37 ± 8.18	
III	29.51 ± 9.75	
**ER status**		
Negative	28.97 ± 10.52	ns
Positive	29.89 ± 8.17	
**PR status**		
Negative	29.17 ± 8.86	ns
Positive	29.92 ± 8.58	
**Her2 status**		
Negative	29.87 ± 8.61	ns
Positive	28.66 ± 8.78	
**DTC status**		
Negative	30.51 ± 8.69	0.004
Positive	28.51 ± 8.54	

*ns* not significant

### LRG-1 is an independent prognostic marker in patients with early breast cancer

Next, we assessed the potential of serum LRG-1 as a prognostic biomarker in patients with early BC. Using maximally rank statistics, the cohort was divided at the optimal cut-off into a LRG-1^high^ and a LRG-1^low^ group (cut-off of 33.64 µg/ml). The mean levels of LRG-1 was the following: LRG-1^high^ group: 40.63 µg/ml (range 33.73–80.70 µg/ml) and LRG-1^low^ group: 25.76 µg/ml (range 9.92–33.63 µg/ml). During the follow-up, 33/133 (24.8%) cases of breast cancer-specific deaths were reported within the LRG-1^high^ group, whereas only 41/368 (11.1%) cases were documented in the LRG-1^low^ group (*p* = 0.0003; odds ratio 2.63; 95%CI: 1.56–4.36). BC-specific survival was significantly reduced for patients within the LRG-1^high^ group compared with the LRG-1^low^ group (HR 3.25; 95%CI: 1.90–5.58; *p* < 0.0001; Fig. 2a). In addition, OS was significantly reduced for patients within the LRG-1^high^ group compared with the LRG-1^low^ group (HR 3.17; 95%CI: 1.86–5.23; *p* < 0.0001; Fig. 2b). Multivariate Cox analyses identified LRG-1 as an independent prognostic marker for breast cancer-specific survival (*p* = 0.001; hazard ratio 2.61; 95%CI: 1.63–4.18; Fig. 2c).

## Discussion

Our investigation identified serum LRG-1 as an independent prognostic marker in patients with early BC at time of diagnosis. These findings are in line with a previous study that assessed tumor tissue expression levels of LRG-1 in patients with early BC. Here, high LRG-1 levels were associated with a decreased disease-free survival (HR 2.090, 95%CI: 1.205–3.625; *p* = 0.009) as well as lymph node metastasis and histological grade [34]. Although LRG-1 did not correlate with tumor grade or lymph node involvement, our findings on circulating levels of LRG-1 are underpinning its relevance as a marker of poor survival in patients with early BC. Similar correlations of high LRG-1 levels in tumor tissue with reduced patient survival were also shown for melanoma, pancreatic and colorectal cancer [20, 22, 24, 26]. The advantage of having identified serum LRG-1 as a prognostic marker is based on the comparatively easy sampling, its incorporation into routine clinical diagnostics and the reduced costs and time.

Our observations may suggest a tumor-promoting role of LRG-1 in early BC. Although the underlying mechanism is not fully understood, studies in leukemia, pancreatic and colorectal cancer cells describe an anti-apoptotic role of LRG-1 by modulating cell cycle factors and activating tumor-promoting signaling pathways [20, 30, 35, 45]. LRG-1 overexpression protects estrogen receptor-positive MCF-7 BC cells from apoptosis while its knockdown sensitizes them to pro-apoptotic stimuli [35]. Another tumor-promoting mechanism of LRG-1 is linked to tumor-related neoangiogenesis. Here, LRG-1 stimulates an aberrant vessel growth by modulating pericyte–endothelial cell interactions; a process that fuels the establishment of a tumor-supporting microenvironment [46]. In patients with gastric cancer, elevated LRG-1 levels positively correlate with local tumor tissue angiogenesis and tumor cell-conditioned medium promotes migration, as well as tube formation of endothelial cells in vitro [18]. LRG-1 was shown to stimulate vascular endothelial growth factor expression in colorectal cancer cells, as well as to activate migration and invasion, both of which are hallmarks of metastasizing cancer cells [22, 24]. In addition, the systemic increase of LRG-1 levels in endothelial cells adjacent to the primary tumor has been recently identified as a driver event for priming the premetastatic niche. This has been linked to the upregulation of prometastatic perivascular cells, which promote the local formation of highly permeable and disorganized capillaries [2, 21].

In summary, pleiotropic roles of LRG-1 in several steps of tumorigenesis have been described in numerous malignancies [2]. LRG-1 is likely to play a role in early BC biology, too, making it a potential novel therapeutic target. To the best of our knowledge, in vivo models of early or advanced BC have not yet been described in LRG-1 knockout mice. Similarly, studies are lacking using genetically manipulated host- or tumor-derived LRG-1 in BC-bearing animal models. One recent study was investigating the effect of a LRG-1-neutralizing antibody in the MMTV-PyMT BC metastasis model. Here, the postsurgical antibody therapy significantly enhanced the median survival of the mice after resection of the primary tumor [21]. Such investigations are highly warranted to understand the role of LRG-1 and its potential molecular and cellular interaction partners in different stages of tumorigenesis, i.e., ranging from precancerous lesions to overt BC metastases in clinically relevant organs, including bone, lungs, or the brain. In murine models of melanoma and lung cancer, genetic silencing of LRG-1 or pharmacological inhibition have shown to both improve tumor vessel function, to slowdown tumor growth and metastasis, and to improve the delivery and efficacy of cytotoxic drugs and immunotherapies including immune check point inhibitors [21, 26, 46, 47].

The cellular source of LRG-1 in our patients remains unclear but it might be possible that several tissue and cell types are able to secrete LRG-1, especially upon the presence of pathological stimuli [46]. Of note, tumor cells of varying malignancies are able to express and secrete LRG-1 [18, 23]. Although not significantly increased, patients who were diagnosed with pT3/pT4 tumors had elevated levels of LRG-1 compared to those with smaller tumors (pT1/pT2), which could be a result of LRG-1 secretion into systemic circulation by growing tumors. Here, tumor cells and the local tumor microenvironment, such as infiltrating immune cells and cancer-associated fibroblasts are potential sources of LRG-1 [2, 19]. Moreover, LRG-1 can be produced within bone tissue [48] and by endothelial cells [7]. However, it remains to be shown whether cellular LRG-1 expression changes at different stages of the primary tumor, by metastatic spread or by local changes of the immune signature in BC. The clinical or molecular significance of the reduced levels of LRG-1 in patients with micrometastasis evidenced by the presence of DTCs remains unclear. Nonetheless, local LRG-1 tissue levels within BC metastases do not necessarily reflect systemic levels. Combining serum analyses with local primary and secondary tumor tissue immunohistochemistry would help to shed more light on these crucial aspects. Likewise, post-translational modifications of LRG-1 might be produced by different cell and tissue types, potentially resulting in distinct prognostic relevance in BC [49]. In addition, in our cohort, few patients developed bone metastasis during the follow-up period. To further clarify the potential role of serum LRG-1 in predicting bone and/or other metastases, future investigations involving high risk populations with BC are needed.

Notably, we observed higher serum LRG-1 levels in older patients (≥ 60 years). This could be attributed to the increasing levels of inflammation in older adults, particularly considering that LRG-1 can be activated by inflammatory cytokines [2, 50]. Furthermore, findings from additional studies not only show a correlation between plasma LRG-1 levels and age, but also with body mass index, cholesterol levels, and endothelial function [51]. Therefore, it is likely that LRG-1 is modulated by metabolic alterations as well. Additionally, in our study, LRG-1 serum levels were significantly elevated in postmenopausal patients. Given that active hormone therapy involving estrogen and progestin in postmenopausal women decreased LRG-1 proteins, this potentially suggest a modulation of LRG-1 by sex hormones [52].

Our study has potential limitations. First, the study lacks an age-matched control group which would allow to compare LRG-1 levels between healthy patients and early BC patients. Likewise, sequential sampling of LRG-1 serum levels at defined time points would provide more insights into the temporal LRG-1 distribution and the impact of tumor resection. This would also strengthen the rationale for LRG-1-targeted therapies and accurate prognosis in individual patients with varying histological and molecular types of BC. For example, neoadjuvant treatment with letrozole reduces gene expression levels of LRG-1 in patients with estrogen receptor-positive BC [53]. However, the large size of the well-characterized cohort, together with the DTC assessment and the detailed and long follow-up are strengths of our study. Nonetheless, this is the first study that identifies serum LRG-1 as an independent prognostic marker in early BC which confirms findings from previous histological analyses of LRG-1 expression levels [34]. Our findings might translate into novel diagnostic approaches because the analysis of serum samples in patients with cancer are more readily available, cost-effective and easily implementable into routine clinical diagnostics.

## Data Availability

The data underlying this article will be shared on reasonable request to the corresponding author.
